# Management of Multisystem Inflammatory Syndrome in Children (MIS-C) in resource limited settings: The Kenyan Experience

**DOI:** 10.1186/s12969-022-00773-9

**Published:** 2022-12-05

**Authors:** Angela Migowa, Pauline Samia, Sean del Rossi, Oliver Ombeva Malande, Jasmit Shah, Chemutai Kenei, Joy Ayaya, Daisy Jeruto, Laura Oyiengo, Laura Lewandowski

**Affiliations:** 1 Department of Paediatrics and Child Health, Aga Khan University, 3rd Parklands Avenue, P.O.BOX 30270 00100 GPO, Nairobi, Kenya; 2 Department of Public Health and Primary Care, Ghent University, Ghent, Belgium; 3 Brain and Mind institute, Aga Khan University, 3rd Parklands Avenue, Nairobi, Kenya; 4 Nakuru County Referral Hospital, Show Ground Road, Nakuru, Kenya; 5 Department of Medicine, Aga Khan University, Nairobi, Kenya; 6 Department of Clinical Medicine and Therapeutics, University of Nairobi, P.O. Box 19676-00202, Nairobi, Kenya; 7 Avenue Hospital, Kisumu-Kakamega Highway, Kisumu, Kenya; 8 Kapsabet County Referral Hospital, Eldoret – Kisumu Highway, Kapsabet, Kenya; 9 United Nations International Children’s Fund Kenya Country Office, United Nations Ave, Nairobi, Kenya; 10 National Institute of Health, 10 Center Drive, Bethesda, MD USA

## Abstract

**Background:**

Since the onset of the recent COVID-19 pandemic, there have been growing concerns regarding multisystem inflammatory syndrome in children (MIS-C). This study aims to describe the clinico-epidemiological profile and challenges in management of MIS-C in low-middle income countries by highlighting the Kenyan experience.

**Methods:**

A retrospective study at the Aga Khan University Hospital Nairobi, Avenue Hospital Kisumu and Kapsabet County Referral Hospital was undertaken to identify cases of MIS-C. A detailed chart review using the World Health Organization (WHO) data collection tool was adapted to incorporate information on socio-demographic details and treatment regimens.

**Findings:**

Twenty children with MIS-C were identified across the three facilities between August 1st 2020 and August 31st 2021. Seventy percent of the children were male (14 of 20). COVID-19 PCR testing was done for five children and only one was positive. The commonest clinical symptoms were fever (90%), tachycardia (80%), prolonged capillary refill (80%), oral mucosal changes (65%) and peripheral cutaneous inflammation (50%). Four children required admission into the critical care unit for ventilation support and inotropic support. Cardiac evaluation was available for six patients four of whom had myocardial dysfunction, three had valvulitis and one had pericarditis. Immunoglobulin therapy was availed to two children and systemic steroids provided for three children. There were no documented mortalities.

**Interpretation:**

We describe the first case series of MIS-C in East and Central Africa. Majority of suspected cases of MIS-C did not have access to timely COVID-19 testing and other appropriate evaluations which highlights the iniquity in access to diagnostics and treatment.

**Supplementary Information:**

The online version contains supplementary material available at 10.1186/s12969-022-00773-9.

## Introduction

The coronavirus disease 2019 (COVID-19) caused by severe acute respiratory syndrome coronavirus 2 (SARS-CoV-2) has led to the ongoing pandemic afflicting individuals of all ages [1] with over 6 million cases diagnosed globally [2]. Though children are less likely than adults to become severely ill, preschool-aged children and infants are more likely than older children to have severe clinical manifestations [2–4].

It is postulated that the COVID-19 virus causes capillary inflammation with complement activation [5, 6]. This leads to multi-organ failure and shock [1, 4, 7]. MIS-C may also present with peculiar abdominal symptoms and elevated inflammatory markers [6]. Laboratory testing has detected positive serology in majority of patients linking COVID-19 as a possible cause of this presentation [1, 4]. Children have responded to anti-inflammatory treatments, including parenteral immunoglobulin, steroids and biological therapies [1, 8, 9].

Given that MIS-C is a rare but potentially fatal condition, more data is required to understand the differences in burden, severity and outcomes of COVID-19 in low-middle income countries compared to high-income countries. It is important to describe the clinical features and outcomes of MIS-C in our context within sub-Sahara Africa. Better characterization of MIS-C will be useful in defining optimal management approaches applicable within our region [10, 11].

Assessment of the rates and long-term effects of MIS-C on children will be important in accurately modelling the pandemic and to ensure that appropriate resources are allocated to children requiring care [2]. Due to the vaccine inequity between high income and low income countries, children in Kenya and similar settings are likely to lag behind in vaccination thus putting them at higher risk of developing MIS-C. Furthermore, research has shown that clinical outcomes of MIS-C among black children are worse compared to other races [12]. Consequently, forecasting resources for COVID-19 alone without adequately planning for possible MIS-C that may follow childhood infections would be inadequate. The data obtained from this study shall contribute towards a better understanding of the MIS-C presentation in our context, response to available therapies and prognosis among these children in low middle income countries.

## Methods

### Study setting

We carried out a retrospective cohort study between 1st August 2020 to 31st August 2021 at three facilities; the Aga Khan University Hospital, Nairobi (AKUHN) (referral facility), Avenue Hospital in Kisumu County (private facility) and Kapsabet County Referral Hospital in Nandi County (referral facility).

Approvals for this study were obtained from the Institutional Ethics Review Committee at the Aga Khan University (*Ref: 2020/IERC-86(v2),* the National Commission for Science, Technology and Innovation-NACOSTI (*NACOSTI/P/20/6232*) and Ministry of Health of Kenya for the participation of the respective institutions.

### Inclusion Criteria

Data from medical records of all patients aged 0–18 years who fulfilled the World Health Organisation (WHO) MIS-C case definition (Additional file 1: Appendix 1) [13]; were included. The following patients were excluded from the study; children with prior cardiac disease, children prior gastro-intestinal disease or prior autoimmune conditions.

Medical records of both paediatric inpatients and outpatients who fulfilled the WHO MIS-C case definition underwent a detailed chart review by a trained recruiter at the health facility and data captured using an adapted WHO data collection tool collected and securely stored on the Research Electronic Data Capture - REDCap platform (Vanderbilt and National Institute of Health) [14]. Following data collection, records were cross checked for completeness and accuracy.

Descriptive statistics were presented where categorical data was presented as frequencies and percentages while continuous data was presented as medians and interquartile ranges. SPSS was used for analysis (IBM Statistical Package for the Social Sciences version 22·00).

## Results

### Demographics

Twenty children were identified to have MIS-C in this study amidst an estimated paediatric population of 24,920,161 [15]. The bio-demographic characteristics are illustrated in Table 1.

**Table 1:** Characteristics and General Demographics of paediatric patients admitted with MIS-C

Variable *n =* 20	No. (%)
Sex	
Male	14 (70.0)
Female	6 (30.0)
Ethnicity	
African	17 (85.5)
Other	3 (15.0)
Age (years) (median [IQR])	3.98 (1.71–7.44)
Family socio-economic status	
Low (GMI < 100,000 ksh)	2 (10.0)
Average (GMI 100,000–500,000 ksh)	12 (60.0)
vHigh (GMI > 500,000 ksh)	6 (30.0)
Years of formal education of parent(s)	
Elementary	1 (5.0)
High School	1 (5.0)
College	11 (55.0)
Unknown / Not reported	7 (35.0)
Type of Health Care	
Public	2 (10.0)
Private	18 (90.0)
Geographic Distribution (County / Town)	
Kapsabet	2 (10.0)
Kisumu	6 (30.0)
Nairobi	12 (60.0)

*MIS-C* Multisytemic Inflammatory Syndrome in Childhood, *GMI* Gross monthly income;

1 USD = 112 Ksh; Ksh-Kenya shillings; USD-United States Dollar

### Clinical Features of MIS-C Patients

Thirty-five percent of MIS-C patients (7 of 20) had respiratory symptoms 4 weeks prior to the onset of MIS-C. Only 10% of the patients (2 of 20) reported a confirmed history of COVID-19, two (10%) had household members with confirmed COVID-19 infection, 6 (30%) had contact with COVID-19 household members. Fifty percent (10 of 20) of the participants had probable MIS-C given that antibody testing was not done despite suspected history of COVID-19 contact. Twenty-five per cent of the children (5 of 20) had a COVID-19 test done of which only one had a positive PCR test and another had a positive antibody test. All the five children tested had a negative COVID − 19 antigen test. None of the children had received prior COVID-19 vaccinations.

Table 2 illustrates the clinical features of the patients at admission and Table 3 highlights the prior co-morbid status of the patients. As regards laboratory investigations, inflammatory markers were elevated with a relatively low neutrophil count. As regards management,90% of the children had haemoglobin test done and 5 children had a positive COVID 19 test (4 positive COVID 19 and 1 positive COVID 19 antibody). Eighty percent of the children received a course of antibiotics while 25% (5 of 20) received immunomodulatory therapy. Four of the 20 patients with MIS-C, required critical care with ventilation support. One patient required inotropic support. Further details of the investigations and management is illustrated in Tables 4 and 5 respectively.

**Table 2:** Clinical features among patients diagnosed with MIS-C at admission

Variable *n =* 20	No. (%)		No. (%)
Fever	18 (90.0)	Chest pain	1 (5.0)
Rash	12 (60.0)	Respiratory Distress	19 (95.0)
Non-purulent Conjunctivitis	4 (20.0)	Abdominal pain	10 (50.0)
Oral mucosal Inflammation	13 (65.0)	Diarrhea	10 (50.0)
Peripheral Cutaneous Inflammation	10 (50.0)	Vomiting	15 (75.0)
Hypotension	2 (10.0)	Headache	1 (5.0)
Tachycardia	16 (80.0)	Seizures	1 (5.0)
Delayed CRT > 2 sec	16 (80.0)	General Malaise	7 (35.0)
Skin mottling	1 (5.0)	Arthritis / Arthralgia	5 (25.0)
Decreased Urine output (< 1 ml/kg/hr)	3 (15.0)	Nasal Congestion	5 (25.0)
		Cough	7 (35.0)

*MIS-C* Multisytemic Inflammatory Syndrome in Childhood;

**Table 3:** Underlying comorbidities among MIS-C patients at admission

Variable *n =* 20	No. (%)		No. (%)
Inflammatory disorders	2 (10.0)	Chronic Kidney Disorders	0 (0.0)
Hypertension / Chronic Cardiac Disease	1 (5.0)	Chronic Neurological Disorders	0 (0.0)
Asthma / Chronic Pulmonary Disease	1 (5.0)	Congenital Immune suppression	1 (5.0)
Malignant Disorders	0 (0.0)	Diabetes/ Endocrine Disorders	0 (0.0)
History of contact with confirmed COVID-19 Infection (COVID-19 PCR positive)	10 (50.0)	Chronic Liver Disorders	0 (0.0)
History of contact with unconfirmed COVID-19 Infection	10 (50.0)	Haematological Disorders	0 (0.0)

*PCR* Polymerase chain reaction

**Table 4:** Laboratory and Imaging findings in paediatric patients diagnosed with MIS-C

Laboratory Markers	Median value (IQR)	Imaging Features		N (%)
Blood cell counts		**Chest X-ray (*n =*** **4)**		
Total white cell counts (×10/L) (*n =* 8)	9.79 (8.06–13.19)	Pulmonary consolidation		2 (50.0)
Neutrophils (×10^9^/L) (*n =* 7)	5.26 (3.94–8.36)	Pleural effusion		2 (50.0)
Lymphocytes (×10^9^/L) (*n =* 7)	3.27 (1.82–518)			
Platelets (×10^9^/L) (*n =* 6)	325.5 (236.0–426.0)	**Echocardiography (*n =*** **6)**		
		Myocardial dysfunction		4 (66.7)
Biochemistry		Pericarditis		1 (16.7)
Procalcitonin (ng/mL) (*n =* 3)	0.17 (0.10–69.52)	Valvulitis		3 (50.0)
CRP (mg/L) (*n =* 16)	68.15 (19.88–143.0)	Coronary abnormalities		2 (33.3)
Haematocrit (%) ((*n =* 16)	34.25 (31.6–36.1)			
Haemoglobin (g/L) (*n =* 18)	11.5 (9.6–12.2)	**Bacterial Blood Culture (*n =*** **20)**		
Creatinine (mmol/L) (*n =* 12)	45.5 (34.0–52.0)	Positive		1 (5.0)
Sodium (mEq/L) (*n =* 12)	134.0 (133.5–137.0)	Negative		19 (95.5)
Potassium (mEq/L) (*n =* 12)	4.3 (3.91–4.97)			
Urea (mmol/L) (*n =* 12)	3.8 (2.24–7.25)	**COVID-19 testing**		
		PCR (*n =* 16)		
ALT (U/L) (*n =* 8)	21.5 (8.5–35.65)	Positive		4 (25.0)
AST (U/L) (*n =* 8)	34.5 (23.5–45.5)	Negative		12 (75.0)
Total Bilirubin (μmol/L) (*n =* 7)	6.0 (5.0–11.0)			
Lactate (mmol/L) (*n =* 1)	2.76 (2.76–2.76)	Rapid antigen test (*n =* 5)		
Albumin (g/dL) (*n =* 8)	34.0 (25.5–41.5)	Positive		0 (0.0)
INR (*n =* 3)	1.25 (0.85–1.3)	Negative		5 (100.0)
		Antibody test (*n =* 1)		1 (100.0)
		Positive		0 (0.0)
		Negative		

*CRP* C-reactive protein, *ESR* Erythrocyte sedimentation reaction, *ALT* Alanine transaminase, *AST* Aspartate transaminase, *INR* International normalized ratio, *PCR* Polymerase chain reaction, *MIS-C* Multisytemic Inflammatory Syndrome in Childhood

**Table 5:** Management and Clinical outcomes of patients diagnosed with MIS-C

Clinical Outcomes	*N =* 20 No. (%)
Drug Therapy	
Antibiotics	16 (80.0)
Intravenous Immunoglobulins	2 (10.0)
Systemic Steroids	3 (15.0)
NSAIDs	5 (25.0)
Non-invasive Oxygen therapy	4 (20.0)
Invasive Ventilation	1 (5.0)
Inotropic support	1 (5.0)
Admission to Intensive Care Unit	4 (20.0)
Discharged Home	20.0 (100%)
Death	0.0 (0.0)

*MIS-C* Multisytemic Inflammatory Syndrome in Childhood, *NSAIDs* Non Steroidal Anti-inflammatory drugs

### Cardiac Complications

Echocardiogram evaluation was done for six children and revealed four had myocardial dysfunction, three had valvulitis and one had pericarditis. There was no documentation of any further follow up except for one child who had normal cardiac evaluation at 6 weeks post discharge. There were no mortalities documented during the period of the study.

## Discussion

We report 20 MIS-C patients across 3 healthcare facilities in Kenya. The median age was 3·98 years (range 1·71–7·44) and 70% of the children were male. Our colleagues in South Africa had a cohort of 68 patients with a much higher median age of 7 years (range 3·6–9·9) [16]. In our cohort, MIS-C was characterized by certain demographic features and clinical presentations including being aged 6 to 12 years, fever (90%), tachycardia (80%), prolonged capillary refill (80%), vomiting (75%), oral mucosal changes (65%) and peripheral cutaneous inflammation (50%). Similarly, in the South African study by Webb and co-workers, the most common clinical features in children with MIS-C were fever (100%), tachycardia (98·5%), rash (85·3%), conjunctivitis (77·9%), abdominal pain (60·3%) and hypotension (60·3%) [16]. Eighty five percent of the children in the South African cohort had gastrointestinal symptoms, including abdominal pain (60·3%), diarrhea (58·8%) and vomiting (10/41) [16].

In the cohort studied by Webb and colleagues, 23% of children with MIS-C (14/61) had a confirmed SARS-CoV-2 contact, 18% (11/61) had a suspected contact and 59% (36/61) had no contact reported [16]. In our cohort only 10% of the patients (2 of 20) reported a confirmed history of COVID-19, 2 (10%) had household members with confirmed COVID-19 infection, six (30%) had household members with suspected COVID 19 infection. The other 10 participants (50%) did not have COVID 19 test done or any known history of COVID 19 infection despite suspected contact. One challenge clinicians face to date in Kenya is that the Government has not authorized COVID 19 antibody testing for routine clinical practice [17] but for research purposes only. Consequently, given the retrospective nature of our study, prior COVID-19 exposure was not routinely assessed in clinical practice. It is important to highlight children exposed to COVID-19 infection can be severely ill with negative COVID-19 PCR and antigen tests thus a high index of suspicion is key in detecting MIS-C cases. The lack of diagnostics in our context might have contributed to the low COVID-19 testing and positivity rate.

There are few reports of MIS-C is sub-Saharan Africa, which may lead to a presumption that this syndrome is not identified in these populations yet low and middle-income countries (LMICs) have the challenge of delayed vaccination, poor vaccine coverage, inadequate diagnostics, sub-optimal management options despite a relatively larger proportion of younger people in their population [16]. In February 2021 the alpha variant was detected in Kenya [18]. The alpha variant has been reported to be less transmissible than other variants such as the delta variant but associated with a higher risk of acute respiratory distress syndrome [19, 20].

In every cohort reported to date, black children are disproportionately affected by MIS-C [1, 2, 13, 14] with an estimation of a nearly six times higher risk of developing MIS-C as compared to caucasian children [16]. In the case series from South Africa by Webb and colleagues for example, they found that black children were over-represented in the MIS-C group (62% vs 37%, *p* = 0·002) [15]. Kenya is a low-middle income country (LMIC), but the centers reporting cases had the resources to identify, treat and document these cases. In rural areas, it is possible that more children remain undiagnosed due to lack of resources and this may explain the low or no detection rate in other county referral hospitals across Kenya further highlighting inequities in resources available to manage these patients.

Odisha and colleagues in eastern state of India analyzed a cohort of 21 children the majority of whom were male (76·2%) and the predominant age group was 6–10 years [16]. However, unlike our cohort which had no mortality reported, Odisha and co-workers reported a mortality of 9% in their cohort which is higher than that reported in western literature [16]. Majority of their cases were positive for severe acute respiratory syndrome coronavirus antibody [16]. Solanki and co-workers in eastern India studied a cohort of 10 children and there was no mortality; findings which are similar to our cohort [17]. Further studies are required to help predict which category of children are likely to develop MIS-C and progress on to have the risk of mortality [17]. This will help us prioritize resources for the paediatric population that truly need it.

As regards laboratory investigations, our cohort revealed patients with MIS-C had markedly elevated inflammatory markers. It is important to note that less than 50% of our cases had the laboratory investigations of interest such as inflammatory markers, white cell count, platelet count, INR, ferritin and electrolytes.

As regards cardiac evaluation in our cohort, 4 patients had myocardial dysfunction, 3 had valvulitis and 1 had pericarditis. There was no documentation of any further follow up except for 1 child who had normal cardiac evaluation at 6 weeks post discharge. The sub-optimal cardiac evaluation may be attributed to the lack of awareness of cardiac complications and skilled personnel to carry out comprehensive cardiac evaluation. On the contrary, in the South African cohort, 71% had cardiac involvement which included pericardial effusions (17·6%), mitral regurgitation (36·8%) and coronary artery aneurysms (5·9%) [16]. The estimated median ejection fraction was 47% (IQR 39,60) [16]. Children with MIS-C and hypotension had a lower EF compared to non-hypotensive patients [16]. In the cohort studied by Fieldstein and co-workers, the most severe cardiovascular involvement from MIS-C, included left ventricular dysfunction and coronary artery aneurysms which resolved within 30 days [21].

In our study treatments offered to the MIS-C patients included; antibiotics *n =* 16 (80·0%), intravenous immunoglobulin (IVIG) *n =* 2 (10·0%), systemic steroids *n =* 3 (15·0%) and non-steroidal anti-inflammatory drugs (NSAIDS) *n =* 5 (25·0%). Among the patients in the South African cohort, IVIG was the most frequently used medication given to 94·1% of the children [16]. Other medications given were intravenous methylprednisolone given to 64·7% of the children and 6% (4/68) of the children received an IL-6 inhibitor (tocilizumab) [16]. In our cohort, patients responded well to IVIG and steroid therapy with no requirement of biological therapies. Nonetheless, these therapies are still beyond the reach of many children who need it due to cost and the monitoring required during their administration.

In our cohort, among the patients admitted to the critical care unit, compensated cardio-respiratory failure was a common feature. Webb et al. found 12 (52%) of the 23 children required admission to an intensive care unit, most commonly due to myocardial dysfunction [12]. On the contrary, Matsubara et al. demonstrated persistent abnormalities in strain and diastolic function in patients with MIS-C and normal ejection fraction [22]. These data, together with literature in adult patients with COVID-19, suggest that subclinical myocardial injury may persist even when traditional measures of left ventricular systolic function are normal [23]. Despite this, only 30% (6 of 20) of our cases had a cardiac assessment further highlighting the gap in clinical are for these patients. Given that there was no systematic cardiac follow up of our cohort. It remains unknown what was the impact on the cardiac health of these children majority of whom did not receive immunomodulatory therapy.

In order to understand the long-term implications for myocardial health, including risk for myocardial fibrosis and diastolic dysfunction, it is critical to have comprehensive assessment of left ventricular systolic and diastolic function in a large, multicenter cohort followed up longitudinally with centralized review of cardiac imaging [23]. Cardiac magnetic resonance imaging and the rare endomyocardial biopsy or postmortem specimen may further help to clarify the underlying pathology and mechanisms of myocardial involvement in MIS-C [23].

We encourage child health professionals to collaborate locally, regionally and internationally in carrying out comparative effectiveness research to determine the most appropriate and feasible treatment modalities and seek biomarkers and other predictors to identify children at high risk of MIS-C and intervene promptly and appropriately to mitigate against premature morbidity and mortality.

Some of the challenges faced in management and conducting research among MIS-C cases in our context is lack of resources for diagnostics and management. One in every 2 children did not have any COVID test done. In our cohort a quarter of the participants had a COVID-19 PCR test done. This low proportion could be attributed to low index of suspicion of COVID, lack of availability of the investigations or the high cost of the tests. Nonetheless in the face of probable MIS-C in our context, one would error on the side of caution and treat even in the absence of confirmatory COVID 19 testing. Similarly, Webb et al. summarized the first 23 cases of MIS-C in South Africa and proving previous COVID-19 disease (or SARS-CoV-2 infection), or likely contact with someone who has had COVID-19, was a limitation in their data because of poor access to SARS-CoV-2 antibody testing and restricted community testing in the region [12]. Most of this cohort had no confirmed or suspected infection or no contact with COVID-19, and no access to antibody tests, but all met clinical diagnostic criteria and had likely community contact with the disease [12]. A high level of suspicion was required during diagnosis because the presenting features of MIS-C were nonspecific (persistent fever, rash, and abdominal pain) [12]. Our case series found that none of our patients had any known potential risk factor other than COVID-19 contact.

Our study had several limitations. First, data collection was retrospective thus there were cases of missing data or incomplete reporting. We mitigated this by training the research personnel at each site that participated in data collection and recording. Second, missing data were not imputed and might be non-random. Third, participating hospitals may not be generalizable and likely overrepresented patients seeking care at tertiary care centers. Fourth, only 20% of patients had echocardiograms, most patients did not have detailed cardiac assessments. Consequently, left ventricular dysfunction and coronary aneurysms could have been underappreciated. Fifth, the efficacies of different immunomodulatory regimens on recovery of cardiac function in the current study were not examined. Sixth, because MIS-C is thought to be delayed in onset after SARS-CoV-2 infection, its distinction from acute COVID-19 could be improved by elucidating the temporal progression from viral exposure to disease onset which we were unable to ascertain given the retrospective nature of the study. Lastly, due to lack of availability of resources, we did not have the capacity to follow up diligently the 47 county referral hospitals to encourage them to enroll participants.

In conclusion, we provide a detailed clinical description of a cohort of children with confirmed and suspected MIS-C cases in Kenya. These data demonstrate that MIS-C occurs in our setting, and can cause serious disease in the paediatric population in our region. Lack of access to care means that the rates in this study are likely lower than the cases nationally. Further studies should be done to discover true case ascertainment, and highlights the case for equity in vaccination efforts for children and adolescents.

### Data sharing

All de-identified data will be available on reasonable request to the corresponding author within a reasonable timeframe.

## Supplementary Information

**Additional file 1.**
