# Supplementary material for: Management of Multisystem Inflammatory Syndrome in Children (MIS-C) in resource limited settings: The Kenyan Experience
Source: Pediatr Rheumatol Online J. 2022 Dec 5;20:110. doi: 10.1186/s12969-022-00773-9 (PMC9721021; doi:10.1186/s12969-022-00773-9)
Supplement: Supplementary file 1 — Additional file 1. [file 12969_2022_773_MOESM1_ESM.docx]

**Appendix 1**

WHO Criteria for Preliminary Case Definition of MISC

**Preliminary case definition[a]**

Children and adolescents 0–19 years of age with fever > 3 days

**AND** two of the following:

1. Rash or bilateral non-purulent conjunctivitis or muco-cutaneous inflammation signs (oral, hands or feet).
2. Hypotension or shock.
3. Features of myocardial dysfunction, pericarditis, valvulitis, or coronary abnormalities (including ECHO findings or elevated Troponin/NT-proBNP),
4. Evidence of coagulopathy (by PT, PTT, elevated d-Dimers).
5. Acute gastrointestinal problems (diarrhoea, vomiting, or abdominal pain).

**AND**

Elevated markers of inflammation such as ESR, C-reactive protein, or procalcitonin.

**AND**

No other obvious microbial cause of inflammation, including bacterial sepsis, staphylococcal or streptococcal shock syndromes.

**AND**

Evidence of COVID-19**(**RT-PCR, antigen test or serology positive), or likely contact with patients with COVID-19.
